# Modeling the window of implantation: insights from endometrial biopsy and menstrual blood-derived organoids and endometrial stromal cells

**DOI:** 10.1093/hropen/hoaf063

**Published:** 2025-10-15

**Authors:** Francesca Paola Luongo, Irene Ortega Baño, Giuseppe Belmonte, Mariangela Gentile, Eugenio Paccagnini, Andres Salumets, Paola Piomboni, Alice Luddi

**Affiliations:** Department of Molecular and Developmental Medicine, University of Siena, Siena, Italy; Department of Molecular and Developmental Medicine, University of Siena, Siena, Italy; Department of Molecular and Developmental Medicine, University of Siena, Siena, Italy; Department of Life Sciences, University of Siena, Siena, Italy; Department of Life Sciences, University of Siena, Siena, Italy; Division of Obstetrics and Gynaecology, Department of Clinical Science, Intervention and Technology (CLINTEC), Karolinska Institute and Karolinska University Hospital, Stockholm, Sweden; Department of Obstetrics and Gynaecology, Institute of Clinical Medicine, University of Tartu, Tartu, Estonia; Celvia CC AS (Competence Centre on Health Technologies), Research Institute, Tartu, Estonia; Department of Molecular and Developmental Medicine, University of Siena, Siena, Italy; Department of Molecular and Developmental Medicine, University of Siena, Siena, Italy

**Keywords:** menstrual blood-derived organoids, menstrual blood-derived endometrial stromal cells, implantation, decidualization, endometrial receptivity, window of implantation, reproductive medicine

## Abstract

**STUDY QUESTION:**

Can menstrual blood-derived organoids (MB-organoids) and human endometrial stromal cells (MB-ESCs) serve as a physiologically relevant, non-invasive model for studying endometrial function and hormonal response?

**SUMMARY ANSWER:**

MB-organoids and ESCs recapitulate key structural and functional features of the endometrium, responding to hormonal stimulation in a manner comparable to biopsy-derived models, supporting their use in reproductive research.

**WHAT IS KNOWN ALREADY:**

Endometrial organoids derived from biopsy samples have provided valuable insights into endometrial physiology and implantation. However, their reliance on invasive tissue sampling limits their clinical and research applications. Menstrual blood contains viable endometrial cells, yet its potential for generating functional three-dimensional (3D) models remains underexplored.

**STUDY DESIGN, SIZE, DURATION:**

This cross-sectional, *in vitro* cell culture study established and characterized 3D-organoids and ESCs derived from menstrual blood, assessing their structural and functional properties as well as their response to hormonal stimulation over a culture period of several weeks. The work was carried out between October 2023 and December 2024, in two European University hospitals.

**PARTICIPANTS/MATERIALS, SETTING, METHODS:**

Menstrual blood samples were collected from healthy fertile donors (*n* = 6). Isolated endometrial cells were cultured using a three-layer gradient system to generate MB-organoids or selected for deriving MB-ESCs. MB-organoids were characterized based on morphological features, including periodic acid–Schiff (PAS) staining for glycogen deposition, scanning electron microscopy (SEM) for pinopode analysis, and immunofluorescence for epithelial (CK8/18) and stromal (vimentin) markers. ESCs were assessed for decidualization by measuring *IGFBP-1* and *ZBTB16* expression after hormonal stimulation, with mifepristone used to terminate progesterone signaling.

**MAIN RESULTS AND THE ROLE OF CHANCE:**

MB-organoids demonstrated structural and functional characteristics similar to biopsy-derived endometrial organoids, including glycogen accumulation and pinopode formation, indicative of endometrial receptivity. Immunofluorescence confirmed the presence of both epithelial and stromal populations as well as glycodelin A production. MB-ESCs underwent decidualization in response to hormonal stimulation, with *IGFBP-1* and *ZBTB16* upregulation, which was suppressed by mifepristone, confirming their functional relevance.

**LIMITATIONS, REASONS FOR CAUTION:**

This *in vitro* culture system models key endometrial features but lacks the complexity of *in vivo* conditions. While menstrual blood derived organoids and ESCs respond to hormonal cues, donor variability and the absence of immune and vascular components limit their physiological relevance. Larger studies and more advanced co-culture systems are needed to improve reproducibility and better replicate the dynamic endometrial environment.

**WIDER IMPLICATIONS OF THE FINDINGS:**

Menstrual blood provides a non-invasive, accessible source for generating functional endometrial models. MB-organoids and MB-ESCs offer promising applications in reproductive medicine, including drug screening, disease modeling, and personalized therapies for endometrial disorders.

**STUDY FUNDING/COMPETING INTEREST(S):**

This work is supported by the Italian Ministry of University and Research—NextGenerationEU PNRR «THE» (Tuscany Health Ecosystem), Spoke 6—Precision Medicine & Personalized Healthcare ECS_00000017, the Estonian Research Council grant no. PRG1076, Swedish Research Council grant no. 2024-02530, Novo Nordisk Foundation grant no. NNF24OC0092384, and Horizon Europe grant NESTOR, grant no. 101120075. The University of Siena Open Access funding partially supported the APC fees. The authors declare no competing interests.

WHAT DOES THIS MEAN FOR PATIENTS? Menstrual blood can provide a simple, non-invasive source of cells from the lining of the uterus, offering a new way to study how the uterus prepares for pregnancy. This is especially important for women facing infertility, repeated implantation failure, or diseases affecting the uterine lining. Traditional approaches rely on invasive biopsies, which are often uncomfortable and difficult to repeat.

In this study, menstrual blood-derived cells were isolated and employed to establish three-dimensional *in vitro* models as well as endometrial cell cultures. These models replicate key features of the uterine lining, including interactions between different cell types, responses to hormones, nutrient storage, and structural changes that make the uterus receptive to an embryo. Our findings show that menstrual blood-derived models are reliable, accessible, and ethically acceptable tools for investigating endometrial biology. They provide the opportunity to monitor uterine receptivity over time, test potential treatments, and better understand implantation problems.

Although still in the research stage, this approach could, in the future, support personalized reproductive medicine and improve outcomes for women with reproductive challenges. Further validation and optimization are needed before these models can be applied in clinical practice, but they represent a promising advance in female reproductive health research.

## Introduction

The study of the endometrium is crucial for understanding the complex processes of implantation and early pregnancy. Endometrial receptivity is a key determinant of successful implantation, which develops during a specific period in the mid-secretory phase known as the window of implantation (WOI) (Brosens *et al.*, 2014). This transient phase is characterized by molecular and structural modifications, including stromal decidualization and the upregulation of key markers critical for embryo attachment and early pregnancy, such as IGFBP-1 and ZBTB16 (Lalitkumar *et al.*, 2007). Disruptions in the WOI can lead to implantation failure and infertility, underscoring the need for experimental models that accurately replicate endometrial physiology and allow for *in vitro* investigations of implantation dynamics (Luddi *et al.*, 2020; Governini *et al.*, 2021). Traditional biopsy-derived two-dimensional (2D) culture systems have provided valuable insights into endometrial function. However, their use is limited by the invasiveness of sample collection and the significant inter-individual variability, which hinders reproducibility and standardization (Passaponti *et al.*, 2023). These challenges highlight the need for advanced *in vitro* models that more closely mimic endometrial physiology while addressing these limitations.

To this end, biopsy-derived three-dimensional (3D) organoid models have emerged as promising alternatives, offering a more physiologically relevant, *ex vivo* platform for studying endometrial function (Boretto *et al.*, 2017; Turco *et al.*, 2017; Luddi *et al.*, 2020; Zhang *et al.*, 2023). These models closely replicate the architecture and cellular composition of the tissues from which they are derived, making them powerful tools for investigating tissue-specific responses to hormonal stimuli (Boretto *et al.*, 2017; Turco *et al.*, 2017; Luddi *et al.*, 2020). Endometrial organoids have shown the ability to respond to hormonal cues, exhibiting key features like glycogen accumulation and the formation of apical protrusions, or pinopodes, which are hallmarks of endometrial receptivity (Melkozerova *et al.*, 2019; Luddi *et al.*, 2020; Cindrova-Davies *et al.*, 2021). These advances highlight the potential of 3D organoid models in reproductive research, offering a more dynamic and physiologically relevant system for studying implantation and early pregnancy. However, despite their advantages, biopsy-derived organoids still rely on endometrial tissue collection, requiring an invasive procedure that limits their accessibility and clinical applicability. This underscores the ongoing need for alternative, non-invasive models that can capture the complexity of endometrial physiology while overcoming the ethical and logistical constraints associated with biopsy-dependent approaches. Menstrual blood (MB) has recently emerged as a non-invasive, abundant source of endometrial stromal cells (ESCs) and other reproductive tissues, making it an ideal candidate for regenerative and reproductive research (Boretto *et al.*, 2019; Fitzgerald *et al.*, 2019). While MB-derived mesenchymal stem cells are well known for their multipotency and regenerative potential (Patel *et al.*, 2008; Alcayaga-Miranda *et al.*, 2015), the use of MB-organoids and MB-derived human ESCs (MB-ESCs) for modeling endometrial physiology and pathology remains less explored. These organoids hold promise for replicating key functional aspects of the endometrium, yet their capacity to fully model endometrial receptivity during the mid-secretory phase is still under investigation. Recent studies have indicated that they exhibit hormonal responsiveness and can develop endometrial-like structures, including pinopodes—hallmarks of endometrial receptivity (Melkozerova *et al.*, 2019; Luddi *et al.*, 2021). However, their ability to accurately capture critical processes such as the WOI and its associated structural and molecular changes remains to be fully characterized (Saadeldin *et al.*, 2024; Shibata *et al.*, 2024), and further research is needed to determine their physiological relevance and potential applications in reproductive medicine, including fertility research and disease modeling. This study aims to establish and characterize MB-organoids and ESCs, evaluating their response to hormonal stimulation. By comparing them with biopsy-derived counterparts, we seek to determine their ability to replicate key endometrial functions, especially receptivity and decidualization. In addition to organoid characterization, we focus on stromal cells isolated from MB, confirming their functional equivalence to biopsy-derived ESCs. Our findings will shed light on hormone-induced molecular changes and offer a non-invasive platform for studying early pregnancy and implantation.

## Materials and methods

### Sample collection

Endometrial tissue samples and MB were collected from women with proven fertility at the University Hospital of Siena (Italy) and South Estonian Hospital (Võru, Estonia). All patients who participated in the study signed the informed consent approved by either the Ethics Committee of Siena University (CEAVSE, Protocol number 18370) or by the Research Ethics Committee of the University of Tartu, Estonia (No. 330M-8), before enrolment. Participants (*n* = 10) were aged between 22 and 37 years. Exclusion criteria included current infections, endocrine disorders, or hormonal treatments within the last 3 months. A complete medical history, physical examination, and transvaginal ultrasound were performed for each participant before sample collection. For the preparation of endometrial organoids, secretory-phase endometrial scratch samples were collected using a disposable endometrial cell sampler, starting from the uterine fundus and moving downward to the internal cervical ostium. MB was collected using a reusable menstrual cup (OrganiCup, AllMatters, Copenhagen, Denmark or MamiCup, Fossalta di Portogruaro, Italy), with the size chosen based on the donor’s age and obstetric history. Proper use of the menstrual cup was explained, and patients were instructed to collect menstrual flow into a sterile 50-ml tube during the heaviest flow days, typically on days 1–2 of menstruation. The sample was collected after overnight use of the cup and provided in the morning.

### Endometrial glandular organoids and stromal cells from endometrial biopsies

Endometrial biopsies were processed to generate glandular organoids, following a validated protocol (Luddi *et al.*, 2020) with some modifications. Briefly, tissues were washed in PBS (S.I.A.L. S.r.l., Rome, Italy) containing penicillin and streptomycin, then cut into 0.5–1 mm³ pieces. Enzymatic digestion was performed in a solution of Dispase II (1.25 U/ml) (Sigma-Aldrich, D4693, St Louis, MO, USA) and collagenase V (0.4 mg/ml) (Sigma, C-9263) in DMEM-F12 with 10% fetal bovine serum (FBS), incubated at 37 °C with gentle shaking for 30–60 minutes. The digested tissue was filtered through 100 μm cell sieves, and the flow-through was filtered sequentially through 70 µm and 40 µm Falcon cell strainers to remove glandular epithelial components. The filtered cells were centrifuged at 300 × g for 10 minutes, collected for stromal cell culture in Advanced DMEM/F12 (S.I.A.L. S.r.l., Rome, Italy) with 10% FBS, penicillin/streptomycin, and L-glutamine (Life Technologies, Carlsbad, CA, USA). Glandular fragments retained on the sieves were backwashed, pelleted by centrifugation, and resuspended in ice-cold Matrigel (Corning, NY, USA) (1:20 ratio). Drops of 20–25 μl of the Matrigel–cell suspension were plated in a 48-well plate and allowed to solidify at 37 °C. Organoids were cultured in organoid Expansion Media (Supplementary Table S1), with medium changes every 2–3 days. Organoids in culture were passaged manually every 7–10 days.

### Endometrial glandular organoids and stromal cells from MB

Organoids were isolated using a protocol adapted from Cindrova-Davies *et al.* (2021), while stromal cells were retrieved using the above-mentioned protocol. Menstrual samples were promptly couriered to the laboratory for processing and were first treated to remove red blood cells. The samples were then centrifuged at 600 × g for 5 minutes and washed several times with PBS. After each wash, the supernatant was discarded, and the cellular material was collected. The resulting sediment was passed through a 100-μm sieve (Corning, 431752) and further rinsed with PBS. The sieve was inverted over a Petri dish, and the remaining cellular debris was backwashed from the sieve membranes, yielding the solid endometrial gland-containing material and the flow-through was filtered sequentially through 70 µm and 40 µm Falcon cell strainers. The filtered stromal cells were centrifuged at 300 × g for 10 minutes, and collected and cultured in Advanced DMEM/F12 with 10% FBS, penicillin/streptomycin, and L-glutamine.

### *In vitro* hormonal treatments

Biopsy-derived and MB-derived organoids and stromal cells passage (P)2–P3 were exposed to hormonal treatments to induce the typical changes associated with the proliferative and secretory phases of the menstrual cycle. To mimic the proliferative phase (pp), cultures were treated with 10^−8^ M estradiol for 2 days. To simulate the mid-secretory phase (msp), cells were then exposed for 4 days to 10^−8^ M estradiol, 10 mM medroxyprogesterone 17-acetate, and 500 µM 8-bromo cAMP (Luddi *et al.*, 2020). For ESC cultures, treatments were carried out in medium containing 2% charcoal-stripped FBS (Gibco, 12676029, Life Technologies, Carlsbad, CA, USA), which allows cell adhesion and growth while minimizing the influence of endogenous hormones. To specifically inhibit the msp response, ESCs were pre-treated with mifepristone (Mif) at concentrations of 5, 10, and 25 µM 1 hour prior to hormone stimulation, as described in previous studies (Lalitkumar *et al.*, 2007; Helmestam *et al.*, 2014; Focarelli *et al.*, 2018). The effectiveness of this treatment in inhibiting decidualization was assessed by measuring *IGFBP-1* mRNA levels, a key marker of this process. As shown in Supplementary Fig. S1, all tested concentrations exhibited an inhibitory effect, with the strongest suppression observed at 10 µM (*P* < 0.001), which was chosen for further experiments. After 72 hours of hormonal treatment, ESCs were trypsinized and pelleted for subsequent analysis. Organoids were collected by washing Matrigel droplets containing the organoids three times with PBS and detaching them using a 1-ml pipette tip to scrape the growth surface of each well. The contents of the wells were centrifuged at 600 × g for 6 minutes to pellet the organoids. Additional washing steps were performed until the Matrigel was completely removed. The organoid pellet was then processed according to the specific analysis requirements.

### Scanning electron microscopy

For SEM, MB-derived organoids were fixed for 2 hours at 4 °C using cold Karnovsky’s fixative, followed by overnight washing in 0.1 M cacodylate buffer (pH 7.2) (Merck, Darmstadt, Germany). The organoids were then post-fixed for 2 hours in 1% buffered osmium tetroxide in veronal acetate buffer and washed again in 0.1 M cacodylate buffer (pH 7.2). After fixation, the organoids underwent dehydration through a graded ethanol series, followed by immersion in tert-butanol and freezing at 0 °C. The tert-butanol was then sublimated in a vacuum chamber to dry the samples. The organoids were sectioned to expose the internal surface, coated with 20 nm gold/palladium, and analyzed using a Quanta 400 (FEI, Hillsboro, OR, USA) scanning electron microscope. Two independent SEM analyses were conducted, with hundreds of organoids fixed and observed by two different operators for each experiment.

### RNA extraction and quantitative (q)RT-PCR analysis

The pelleted MB and biopsy-derived ESCs were resuspended in 300 µl of RNA lysis buffer (RTL) lysis buffer containing β-mercaptoethanol (Qiagen, Hilden, Germany). The samples were then centrifuged at 13 000 g for 1 minute at 4 °C, and the supernatants were processed for RNA extraction using the QIAcube (Qiagen) automated extractor, following the manufacturer’s protocol. RNA was then eluted in 30 μl of water and its concentration and purity were assessed using a NanoDrop spectrophotometer (Thermo Fisher Scientific, Waltham, MA, USA). After a DNase treatment, RNA was reverse transcribed into cDNA, using the iScript gDNA Clear cDNA Synthesis Kit (Bio-Rad Laboratories, Hercules, CA, USA), according to the manufacturer’s instructions. Gene expression was evaluated in triplicate by qRT-PCR on a CFX connect Real-Time PCR Detection System (Bio-Rad Laboratories) using SsoFast EvaGreen Supermix (Bio-Rad Laboratories). The gene-specific primer sets used are listed in Supplementary Table S2. Melting curve analysis was performed to confirm product specificity. To normalize gene expression in stromal cells and organoids, the mRNA expression levels of the housekeeping genes *HPRT1* and *GAPDH* were measured in all samples. The latter were selected as the most appropriate housekeeping genes for our experiments; the expression levels obtained from each sample of four reference genes were analyzed through the RefFinder tool (https://github.com/fulxie/RefFinder). Gene expression changes were calculated using the 2^−ΔΔCt^ method.

### Immunofluorescence analysis

MB and biopsy-derived organoids were resuspended and fixed in 10% neutral buffered formalin at room temperature for 24 hours. Following fixation, 2–3 organoids were embedded in a drop of 4% agar and allowed to solidify. The agar-embedded organoids were then paraffin-embedded and sectioned at a thickness of 4 µm. Sections were mounted on Superfrost Plus slides (Thermo Fisher Scientific). For immunofluorescence staining, sections were deparaffinized using xylene and dehydrated through a graded ethanol series. Antigen retrieval was performed by incubating the slides in 10 mM citrate buffer (pH 6.0) at sub-boiling temperature for 20 minutes, followed by cooling at room temperature for 10 minutes. Non-specific binding was avoided by incubating the sections in a blocking solution containing 5% normal goat serum and 1% bovine serum albumin in PBS for 1 hour at room temperature. The sections were then incubated with primary antibodies according to the manufacturer’s instructions and a published protocol (see Supplementary Table S3 for details) (Luongo *et al.*, 2023). Specificity of immunostaining was confirmed by omitting the primary antibody or using unrelated antibodies as negative controls. After primary antibody incubation, slides were washed in PBS, and bound antibodies were detected using Alexa Fluor 488- or 568-conjugated secondary antibodies (Supplementary Table S3). Following three PBS washes, slides were mounted using ProLong Gold Antifade Mountant with 4′,6-diamidino-2-phenylindole (DAPI) (Life Technologies, Carlsbad, CA, USA) for nuclear counterstaining. Imaging was performed using a Leica DMB 6000 microscope equipped with a CFTR6500 digital camera (Leica Microsystems GmbH, Wetzlar, Germany). Images were captured and analyzed using appropriate software.

### Statistical analysis

Statistical analyses were conducted using GraphPad Prism version 9.0 (GraphPad Software, San Diego, CA, USA). Experiments were performed in biological replicates using samples from at least three independent donors. Data normality was assessed based on standardized skewness and kurtosis values. Depending on data distribution, either a Student’s t-test or a Wilcoxon signed-rank test was applied. Comparisons between groups were performed using a one-way ANOVA followed by a Bonferroni post-hoc test. Statistical significance was defined as *P *< 0.05.

## Results

### Organoid formation and structure: comparison between biopsy and MB-derived organoids

**Figure 1.**
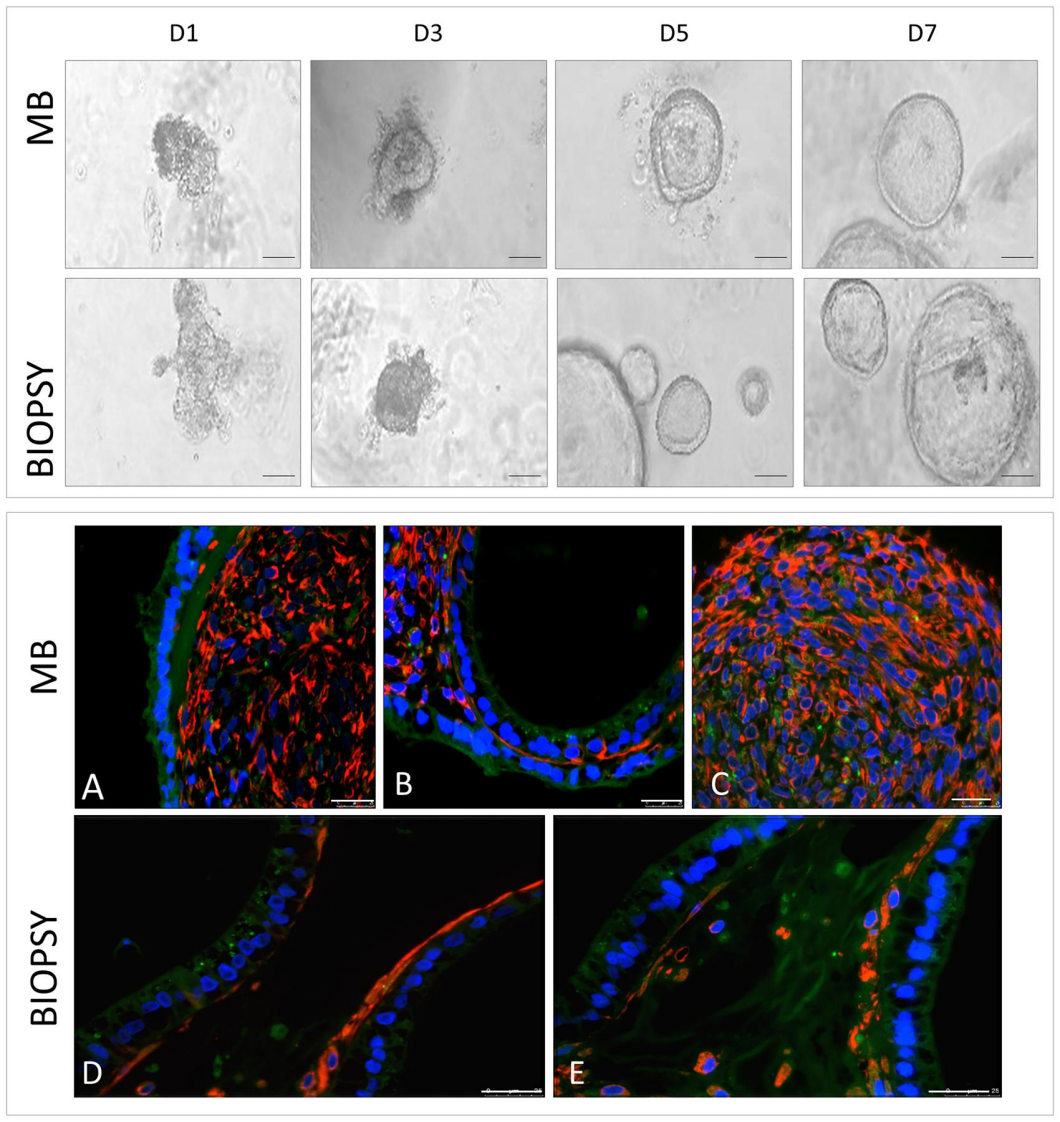
**Morphology and epithelial–stromal composition of menstrual blood- and biopsy-derived organoids.** Upper panel: Brightfield micrographs of organoid cultures from menstrual blood (MB) (*n* = 3) and biopsy (*n* = 3) on different days (D) after plating: D1, D3, D5, and D7. Scale bars, 20 μm. Lower panel: Immunofluorescence staining of vimentin (red) and cytokeratin 8/18 (green) of both MB- (**A–C**) (*n* = 3) and biopsy-derived (**D and E**) (*n* = 3) organoids. Scale bar, 25 μm.

Immediately after receiving the samples from the donors, cells were processed according to the different sources (Biopsy or MB). Cells were plated in Matrigel and observed at various times (days 1, 3, 5, and 7) to monitor and compare organoid development and progression (Fig. 1, upper panel). Organoids from both sources showed no phenotypical development differences during the time frame. While the initial yield of tissue fragments from MB was generally lower and required longer processing than from biopsies, the rate of organoid establishment was comparable to that from endometrial biopsies, confirming previously published data (Cindrova-Davies *et al.*, 2021). To further characterize the cell composition of organoids, immunofluorescence analysis was performed to localize vimentin, a stromal cell marker, and cytokeratin 8/18, an epithelial cell marker. This analysis was conducted on early-passage organoids (P2–P3) derived from both MB and biopsy sources, cultured without hormonal stimulation (Fig. 1, lower panel). Organoids from MB exhibited an external layer of epithelial cells (green), while the internal compartment predominantly consisted of stromal cells (red) (Fig. 1, lower panel, A-C). Moreover, the endometrial biopsy organoids contained epithelial and stromal cell types, underscoring that both MB- and biopsy-derived organoids resemble the *in vivo* endometrial environment (Fig. 1, lower panel, D-E).

### Organoids from MB respond to hormonal stimulation, mimicking the phases of the menstrual cycle

**Figure 2.**
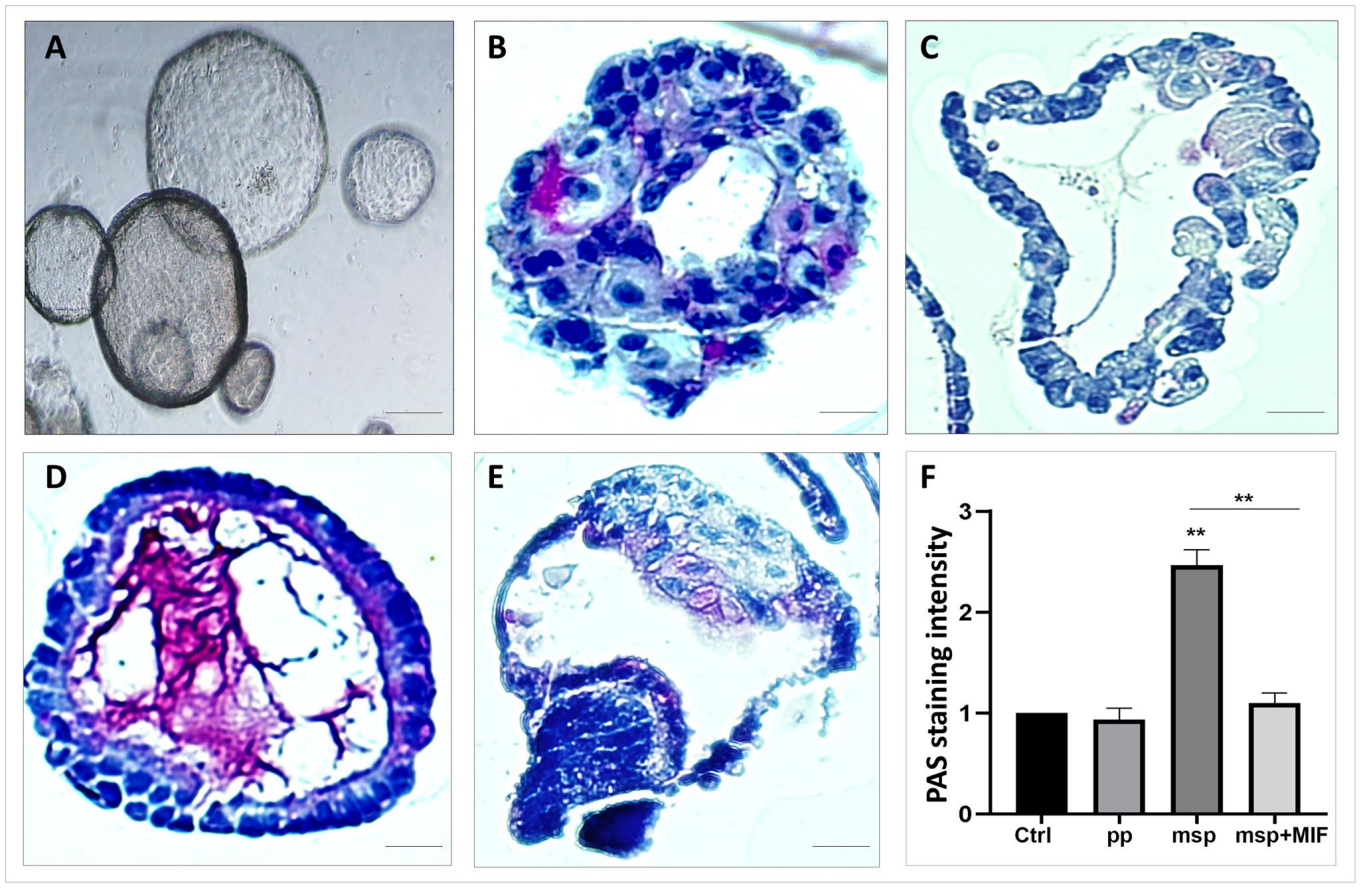
**Glycogen accumulation in menstrual blood-derived organoids under different hormonal treatments.** (**A**) Bright field representative image of menstrual blood (MB)-derived three-dimensional (3D)-organoid cultures. Scale bar, 250 μm. (**B–E**) Periodic acid–Schiff reagent (PAS) staining for glycogen detection in organoids of MB-derived 3D-organoid cultures. (B) Organoids untreated control (ctrl), (C) treated with estrogen (E2) (proliferative phase, pp), (D) with E2, progesterone (P4), cAMP (mid secretory phase, msp), (E) with E2, P4, cAMP and 10 µM mifepristone (msp+MIF). Images are representative of three glandular epithelial organoid preparations. PAS staining appears as scattered red to magenta particles in the cytoplasm. Scale bars: B, 40 μm; C–E, 25 μm. (**F**) Bar chart graph showing the quantification of PAS staining intensity. Data are presented as mean ± SEM of relative fold change (*n* = 3), **P < 0.01. Ordinary one-way ANOVA followed by a Bonferroni post-hoc test.

The human endometrium undergoes specific morphological and molecular transformations in response to the ovarian hormones estrogen (E2) and progesterone (P4) (Dias Da Silva *et al.*, 2024). To model this process *in vitro*, we exposed MB-derived organoids (Fig. 2A) to hormone treatments, using E2 alone to mimic the pp and a combination of E2, P4, and cAMP to induce the msp. As in the case of biopsy-derived organoids (Luddi *et al.*, 2020), immunohistochemical analysis confirmed that MB organoids retained the original histological features of epithelial tissue, exhibiting monostratified or pluristratified columnar epithelium arranged to form glandular structures. Periodic acid–Schiff reagent (PAS) staining validated the functional properties of the MB-derived organoids, resembling those of endometrial glands *in vivo* (Fig. 2B–E). Specifically, we demonstrated that glycogen—a key marker of glandular endometrium—is secreted by the epithelial cells within the msp MB-organoids (Fig. 2D), mirroring the secretory function of endometrial glands observed *in vivo* (Zhang *et al.*, 2024). Notably, this glycogen secretion was inhibited by MIF treatment (Fig. 2E), a progesterone modulator that competes with progesterone for binding to the progesterone receptors in the ligand-binding domain (Papp *et al.*, 2000). The quantitative analysis of PAS staining intensity showed a significant increase in msp-organoids, which was notably reduced by MIF (Fig. 2F).

**Figure 3.**
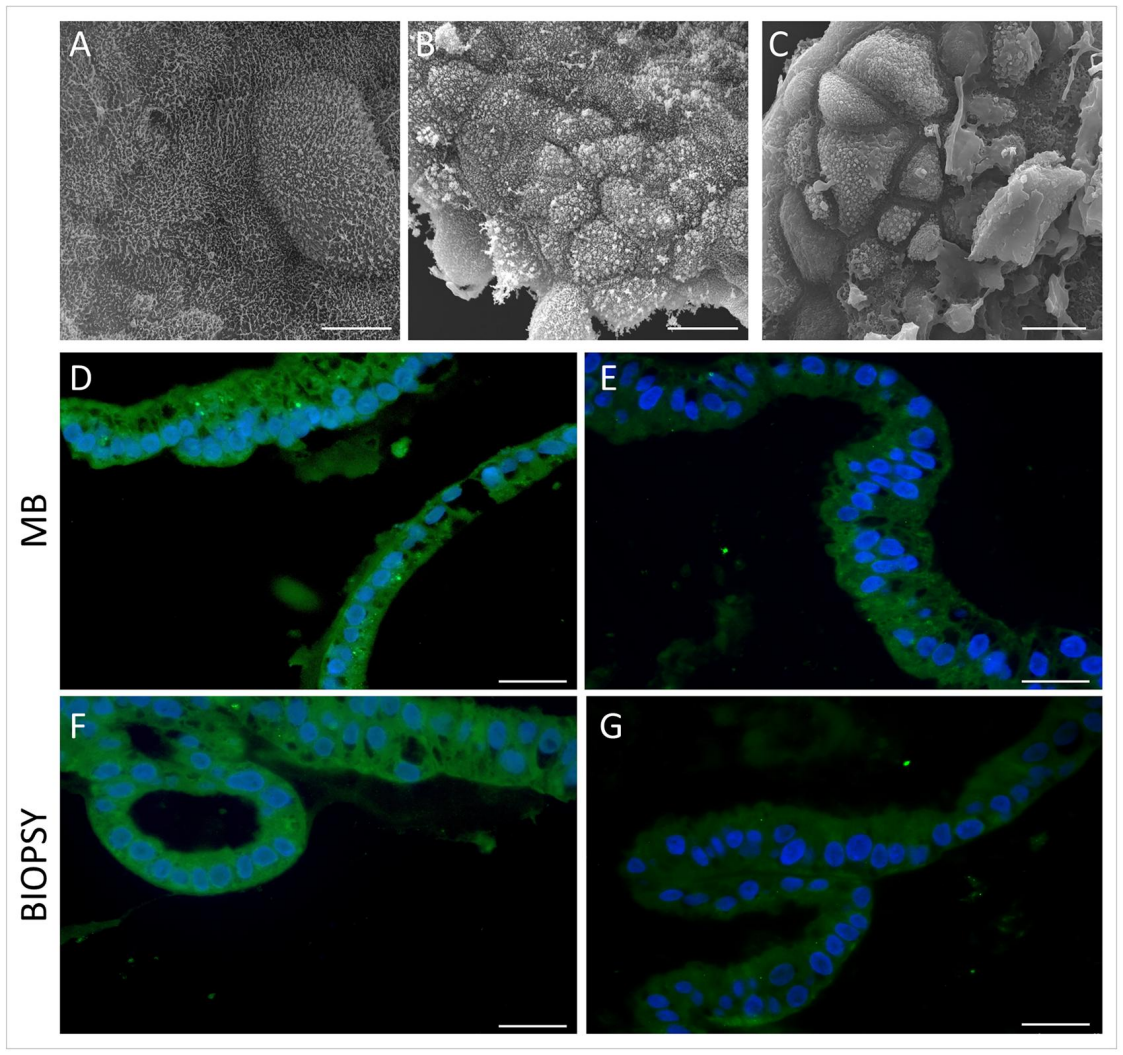
**Surface ultrastructure and glycodelin A expression in menstrual blood- and biopsy-derived organoids during proliferative and secretory phases.** (**A–C**) Scanning electron microscopy of menstrual blood (MB)-organoids under different conditions: (A) untreated control, (B) treated with estrogen (E2) to mimic the proliferative phase, showing numerous microvilli and expanded cilia on the apical surface, and (C) treated with E2 + progesterone (P4) + cAMP to mimic the mid-secretory phase, displaying prominent pinopodes across the surface. These large cytoplasmic protrusions are associated with a significant reduction in microvilli and cilia. (**D–G**) Immunofluorescence staining of glycodelin A (GdA, green) in MB-derived (D and E) and biopsy-derived (F and G) organoids. Proliferative phase (D and F) and mid-secretory phase (E and G) organoids are shown. Nuclei were counterstained with DAPI (blue). Scale bars, 25 μm. Images are representative of three replicates.

We further conducted a SEM analysis to confirm the substantial changes at the surface following various hormone treatments we previously reported for biopsy-derived organoids (Luddi *et al.*, 2020). In untreated MB-derived organoids, the luminal surface is relatively smooth, or it exhibits irregular cytoplasmic micro-extensions (Fig. 3A); MB-organoids treated with E2 to mimic the pp show numerous microvilli and cilia expansions of different lengths from the apical surface of epithelial cells (Fig. 3B). The surface of endometrial organoids mimicking the msp is characterized by the presence of large apical cytoplasmic protrusions traditionally called ‘pinopodes’ (Fig. 3C). The cellular localization of glycodelin A (GdA), a well-established marker of endometrial receptivity, was further evaluated by using immunofluorescence. The analysis revealed positive staining in all samples, with a clear and diffuse signal consistent with a cytoplasmic distribution (Fig. 3D–G). Notably, GdA expression appeared to be influenced by hormonal stimulation, as msp-organoids (Fig. 3E and G) exhibited significantly higher signal intensity compared to pp-organoids (Fig. 3D and F), consistent with previous findings in biopsy-derived organoids (Luddi *et al.*, 2020).

### Gene expression profiling of decidualization-related genes in ESCs from MB

**Figure 4.**
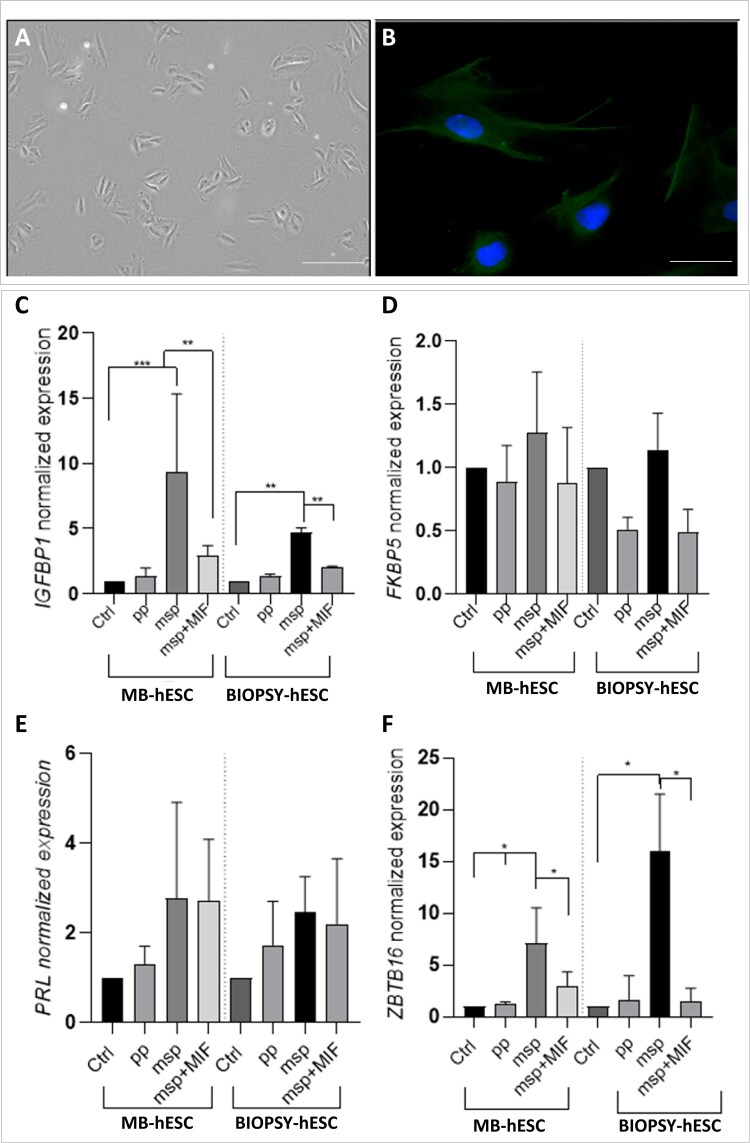
**Analysis of lineage-specific markers and decidualization in menstrual blood- and biopsy-derived endometrial stromal cells.** (**A**) Representative bright-field image of endometrial stromal cells (ESCs) in culture and (**B**) immunofluorescence image showing vimentin (green) and nuclei (blue) in menstrual blood (MB)-derived ESCs. (A) Scale bar, 100 μm. (B) Scale bar, 25 μm. (**C–F**) Gene expression analysis of decidualization markers in MB-derived (*n* = 5) and biopsy-derived ESCs (*n* = 5). The relative mRNA expression levels (2^-ΔΔCt^) of *IGFBP-1* (C), *FKBP5* (D), *PRL* (E), and *ZBTB16* (F) were assessed under the same experimental conditions: vehicle control (Ctrl), treated with estrogen (E2) proliferative (pp), with E2, progesterone (P4), cAMP mid secretory phase (msp), and with E2, P4, cAMP and 10 µM mifepristone (msp+MIF). The dotted grey line separates menstrual blood-derived from biopsy-derived samples. Statistical significance is indicated as *P < 0.05, **P < 0.01, and ***P < 0.001. Data are presented as mean ± SEM of relative fold change.

Building on our findings that MB-derived organoids, like biopsy-derived ones, are predominantly composed of stromal cells (ESCs) in their internal compartment (Fig. 1A–C), we focused on this specific cell type isolated from MB to confirm that their characteristics are comparable to those isolated from endometrial biopsy. As shown in Fig. 4B, all cells exhibited positive vimentin staining, confirming both their stromal origin and the purity of the isolated population. We further explored the characteristics of MB-derived ESCs during the decidualization process—a key step in establishing endometrial receptivity—by comparing them to biopsy-derived ESCs. Similar to MB-derived organoids, we modeled decidualization *in vitro* by priming cells with E2 for 2 days to mimic the pp (ESC-pp), followed by stimulation with E2-P4 and cAMP to simulate the msp (ESC-msp). To block the decidualization process, ESCs were pretreated with 10 µM Mif. Using this setup, we analyzed the expression of key genes involved in decidualization, like *IGFBP-1, FKBP5, PRL*, and *ZBTB16*. Notably, MB-derived ESCs exhibited a gene expression profile in response to hormonal treatment that was consistent to biopsy-derived ESCs. In both cell types, *IGFBP-1* expression was significantly upregulated in the ESC-msp group compared to both vehicle and ESC-pp groups (*P* < 0.001) and was significantly reduced by MIF pretreatment (*P < *0.01) (Fig. 4C). Similarly, *ZBTB16* expression was significantly upregulated following hormonal stimulation to induce msp (*P* < 0.05) in both MB- and biopsy-derived cells, with this effect being efficiently reversed by MIF (*P* < 0.05) (Fig. 4F). Although *FKBP5* and *PRL* also showed increased expression in the ESC-msp samples, this increase was not statistically significant, nor did MIF treatment lead to a significant change in their expression levels (Fig. 4D and E).

## Discussion

This study demonstrated that MB-derived organoids and ESCs provide a reliable and physiologically relevant platform for studying endometrial function. Our findings indicate that organoids derived from MB and endometrial biopsies are morphologically indistinguishable, despite a generally lower initial yield from MB samples. This observation suggests that the organoid-forming cells represent a viable and proliferative subpopulation rather than apoptotic or non-viable cells shed during menstruation. The comparable timing and efficiency of organoid establishment in both sources reflect a natural selection process favoring healthy, functional cells within the culture environment. Our results demonstrate that MB-derived organoids retain the structural and functional characteristics of endometrial glands and respond to hormonal stimuli like biopsy-derived organoids. Additionally, ESCs derived from MB undergo decidualization comparably to those obtained from endometrial biopsy, as evidenced by changes in the expression of key markers such as *IGFBP-1* and *ZBTB16*.

Our data confirm that MB-derived organoids share fundamental characteristics with their biopsy-derived counterparts, including their ability to respond to hormonal stimulation, as previously reported (Cindrova-Davies *et al.*, 2021). PAS staining revealed glycogen accumulation in hormone-stimulated MB-organoids, confirming their secretory function, which is characteristic of the mid-secretory phase. SEM further demonstrated the formation of apical cytoplasmic protrusions, namely pinopodes, in these organoids, a well-established marker of endometrial receptivity (Melkozerova *et al.*, 2019). These findings demonstrate the ultrastructural capability of both MB- and biopsy-derived organoid models to respond to various stimuli, effectively mimicking the menstrual cycle. This highlights the physiological relevance of MB-derived organoids as a non-invasive *ex vivo* model for studying the menstrual cycle and implantation process. Our study also sheds light on the cellular composition of MB-derived organoids. Immunofluorescence analysis revealed both epithelial (CK8/18-positive) and stromal (vimentin-positive) cell populations within the organoid structure. While previous studies have primarily characterized endometrial organoids as epithelial structures (Boretto *et al.*, 2017; Turco *et al.*, 2017), our findings align with recent reports suggesting the presence of stromal components in early-passage organoids (Zhang *et al.*, 2023). This discrepancy may be attributed to differences in culture conditions or passage number, as stromal cells are known to have limited survival in Matrigel-based cultures (Arnold *et al.*, 2001). Notably, hormonal stimulation appeared to influence GdA expression, as msp-organoids displayed significantly higher signal intensity than pp-organoids. This observation aligns with previous findings in biopsy-derived organoids (Luddi *et al.*, 2020). *In vivo*, GdA is secreted by endometrium and pregnancy decidua in response to progesterone, hCG and relaxin (Seppälä *et al.*, 2002). Moreover, specific isoforms of GdA were demonstrated to be differentially expressed during the different phases of the menstrual cycle (Focarelli *et al.*, 2018).

Finally, the presence of pinopodes, known as specific markers of the implantation window (Melkozerova *et al.*, 2019), is an important and novel feature of the hereby described *ex vivo* endometrial model. These data demonstrated the ultrastructural capability of the organoids model, whether from MB or biopsy, to respond to different stimuli to mimic the menstrual cycle.

Decidualization is a critical process for implantation, marked by significant transcriptomic and morphological changes in ESCs (Brosens *et al.*, 2014). In our study, ESCs derived from both MB and biopsy tissues exhibited a similar gene expression profile following hormonal stimulation. Notably, *IGFBP-1*, a well-established marker of decidualization, was significantly upregulated in ESC-msp compared to ESC-pp and vehicle controls. The progesterone receptor antagonist mifepristone significantly inhibited *IGFBP-1* expression, confirming its role in blocking the decidualization process (Lalitkumar *et al.*, 2007; Helmestam *et al.*, 2014). Similar trends were observed for *ZBTB16*, a key transcription factor involved in decidual transformation. Our results align with previous studies that have demonstrated the role of IGFBP-1 and ZBTB16 in decidualization (Lucas *et al.*, 2020). Interestingly, *FKBP5* and *PRL*, additional markers of decidualization, were upregulated following hormonal stimulation, but their expression was not significantly affected by mifepristone. This suggests that *FKBP5* and *PRL* may be regulated by alternative pathways or may require prolonged progesterone exposure for full suppression (Gellersen *et al.*, 2010).

The comparable response of MB-derived and biopsy-derived ESCs to hormonal cues highlights the potential of menstrual blood as a non-invasive source of ESCs for research. Previous studies have demonstrated that MB-derived mesenchymal stem cells retain regenerative potential and multipotency (Patel *et al.*, 2008; Alcayaga-Miranda *et al.*, 2015). Our findings extend this knowledge by demonstrating that MB-derived ESCs are functionally competent in the context of decidualization, further supporting their use in *in vitro* models for embryo implantation and early pregnancy. Previous studies (Laird *et al.*, 1993; Licht *et al.*, 2001; Perrier d’Hauterive *et al.*, 2004) have shown that hCG not only supports corpus luteum function but also acts in a paracrine manner on the endometrium by modulating cytokine secretion. Specifically, hCG appears to increase the production of LIF, a pro-implantation cytokine, while reducing IL-6 secretion, which is known for its pro-inflammatory properties. Although our study, focused on endometrial organoids and stromal cells, did not directly assess cytokine secretion or the modulatory effects of hCG on IL-6, we acknowledge the importance of this mechanism in regulating the immune environment during implantation. The ability of hCG to inhibit IL-6 may contribute to the crucial switch from a pro-inflammatory to a tolerogenic state necessary for successful implantation and pregnancy maintenance. Future studies investigating IL-6 and other cytokine profiles in response to hCG, possibly in combination with progesterone, will be valuable to better understand the delicate balance between inflammation and immune tolerance in the human endometrium.

Building on our findings, this study demonstrates that MB-derived organoids and ESCs provide a reliable and physiologically relevant platform for studying endometrial function. These models successfully replicate key aspects of the menstrual cycle and the decidualization process, making them valuable tools for drug screening, disease modeling, and personalized medicine. Menstrual blood, being a non-invasive, easily accessible, and abundant source of endometrial cells, provides a significant advantage for reproductive health research. However, while these models hold great potential, some limitations must be considered. They may not fully capture the complexity of the *in vivo* endometrial environment, and the inherent variability in MB samples could influence reproducibility. We acknowledge that while we included samples from multiple donors, inherent donor-to-donor variability, such as hormonal background, and genetic factors, may influence organoid behavior and decidualization responses. This variability reflects biological reality but may also affect reproducibility. Second, although MB-derived organoids provide both epithelial and stromal components, our current model does not incorporate immune or vascular elements, which play critical roles in endometrial physiology and pathology. Integrating these components in future work will be essential to more fully recapitulate the *in vivo* endometrial microenvironment. These limitations should be considered when interpreting our findings, especially regarding translational applications. In conclusion, despite some limitations, biopsy and MB-derived organoids and ESCs represent a promising approach for investigating implantation failure and early pregnancy disorders. These models offer valuable insights into reproductive health and have significant potential for advancing drug screening, disease modeling, and personalized medicine. Future research should focus on enhancing the complexity of these models, evaluating their long-term stability, and expanding their applications in reproductive medicine, particularly in personalized treatments and fertility interventions.

## Supplementary Material

hoaf063_Supplementary_Data

## Data Availability

Data are available on request.
