# Supplementary material for: Modeling the window of implantation: insights from endometrial biopsy and menstrual blood-derived organoids and endometrial stromal cells
Source: Hum Reprod Open. 2025 Oct 15;2025(4):hoaf063. doi: 10.1093/hropen/hoaf063 (PMC12596476; doi:10.1093/hropen/hoaf063)
Supplement: hoaf063_Supplementary_Data [file hoaf063_supplementary_data.zip › Supplementary Figure S1-post adjudication clean.pdf]

Supplementary Figure S1

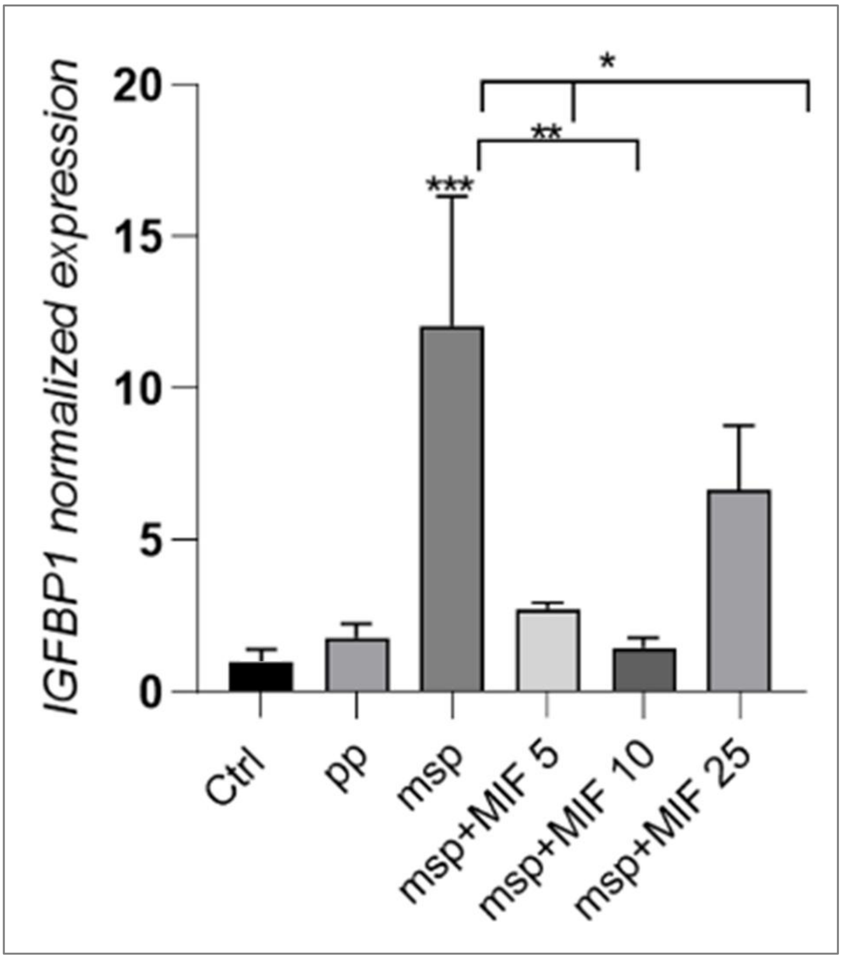

Supplementary Figure S1: *IGFBP1* gene expression in menstrual blood (MB)-derived endometrial stromal cells (ESCs) (n=3) under hormonal stimulation and mifepristone pretreatment. Relative mRNA expression levels of *IGFBP1* were measured by quantitative PCR ($2^{-\Delta\Delta Ct}$ method) under the following conditions: vehicle control (Ctrl), treatment with estradiol alone (proliferative phase, pp), combined treatment with estradiol, progesterone, and cAMP (mid secretory phase, msp), and msp with pretreatment using mifepristone (MIF) at 5, 10, and 25 µM, administered one hour before hormone stimulation. Data are shown as mean ± SEM of relative fold change compared to control. Ordinary one-way ANOVA followed by a Bonferroni post-hoc test. Statistical significance is indicated as *$p < 0.05$, **$p < 0.01$, ***$p < 0.001$.
