# Supplementary material for: Modeling the window of implantation: insights from endometrial biopsy and menstrual blood-derived organoids and endometrial stromal cells
Source: Hum Reprod Open. 2025 Oct 15;2025(4):hoaf063. doi: 10.1093/hropen/hoaf063 (PMC12596476; doi:10.1093/hropen/hoaf063)
Supplement: hoaf063_Supplementary_Data [file hoaf063_supplementary_data.zip › Supplementary Table S2.docx]

**Supplementary Table S2:** Primer list for Gene Expression analysis

| **Target Genes** | **Acronym** | **Sequences** |
| --- | --- | --- |
| *FK506 binding protein 5* | *FKBP5* | FW-TCT CTT TGG GCA GAG CGG AA  RV-TCT AGC CTT CTG CAG CGT GG |
| *Insulin-like growth factor-binding protein-1* | *IGFBP-1* | FW-CTG CTG GTG CGT CTA CCC TT  RV- AGT TGG GGT CTC CCC TGA TCT |
| *Prolactin* | *PRL* | FW- TTCGAGACCTGTTTGACCGC  RV- TCTTTTTGATTCATCATCTGTTGGGC |
| *Zinc Finger and BTB Domain Containing 16* | *ZBTB16* | FW- CAG CCG AGT CCA GCA TCT CA  RV- CTG CTC GGC ACT CTC CTC TC |
| **Target Genes** | **Acronym** | **Sequences** |
| *Hypoxanthine-Guanine Phosphoribosyl Transferase 1* | *HPRT1* | dHsaCPE5192871 |
| *Glyceraldehyde-3-phosphate dehydrogenase* | *GAPDH* | Hs02706624_g1 |
