# Supplementary material for: Modeling the window of implantation: insights from endometrial biopsy and menstrual blood-derived organoids and endometrial stromal cells
Source: Hum Reprod Open. 2025 Oct 15;2025(4):hoaf063. doi: 10.1093/hropen/hoaf063 (PMC12596476; doi:10.1093/hropen/hoaf063)
Supplement: hoaf063_Supplementary_Data [file hoaf063_supplementary_data.zip › Supplementary-Table-S1-ost adjudication clean.docx]

**Supplementary Table S1:** Expansion medium (ExM) composition for the culture of menstrual blood (MB)-Organoids and Biopsy Organoids and hormones and molecules used in the study

| **Expansion medium (ExM) composition for the culture of MB-Organoids and Biopsy Organoids** | | | | | | |  |
| --- | --- | --- | --- | --- | --- | --- | --- |
| **Product** | **Company** | | **Product Number** | | **Final Concentration** | |  |
| DMEM/Ham's F12 | SIAL srl  Rome, Italy | | 12-XHA | | 1X | |  |
| N1 supplement | Merck  Darmstadt, Germany | | N6530 | | 1X | |  |
| B27 supplement minus vitamin A | Life Technologies  Carlsbad, CA,USA | | 12587010 | | 1X | |  |
| Normocin | Invivogen  San Diego, CA, USA | |  | | 50mg/ml | |  |
| N-Acetyl-L-cysteine | Sigma-Aldrich  St. Louis, MO, USA | | A9165-5G | | 1.25 mM | |  |
| L-glutamine | Life Technologies  Carlsbad, CA,USA | | 25030-024 | | 2 mM | |  |
| Recombinant human EGF | Peprotech  Cranbury, NJ, USA | | AF-100-15 | | 50 ng/ml | |  |
| Recombinant human Noggin | Peprotech  Cranbury, NJ, USA | | 120-10c | | 100 ng/ml | |  |
| Recombinant human Rspondin-1 | Peprotech  Cranbury, NJ, USA | | 120-38 | | 500 ng/ml | |  |
| Recombinant human FGF-10 | Peprotech  Cranbury, NJ, USA | | 100-26 | | 100 ng/ml | |  |
| Recombinant human HGF | Peprotech  Cranbury, NJ, USA | | 100-39 | | 50 ng/ml | |  |
| ALK-4, -5, -7 inhibitor, A83-01 | System Biosciences  Palo Alto, CA, USA | | ZRD-A8-02 | | 500 nM | |  |
| Nicotinamide | Sigma-Aldrich  St. Louis, MO, USA | | N0636 | | 10 nM | |  |
| **Hormones and molecules used in the study** | |  | |  | |  | |
| Product | Company | | Product Number | | Final Concentration | |  |
| Estradiol (E2) | Sigma-Aldrich  St. Louis, MO, USA | | E4389 | | 10 nM | |  |
| Medroxyprogesterone 17-acetate (MPA) | Sigma-Aldrich  St. Louis, MO, USA | | M1629 | | 1O mM | |  |
| 8-Bromo-cAMP | Sigma-Aldrich  St. Louis, MO, USA | | A9501 | | 500 µM | |  |
| Mifepristone | Sigma-Aldrich  St. Louis, MO, USA | | M8046 | | 5, 10, 25 µM | |  |
