# Supplementary material for: Modeling the window of implantation: insights from endometrial biopsy and menstrual blood-derived organoids and endometrial stromal cells
Source: Hum Reprod Open. 2025 Oct 15;2025(4):hoaf063. doi: 10.1093/hropen/hoaf063 (PMC12596476; doi:10.1093/hropen/hoaf063)
Supplement: hoaf063_Supplementary_Data [file hoaf063_supplementary_data.zip › Supplementary-Table-S3-post adjudication clean.docx]

**Supplementary Table S3**. List of Antibodies

| *Antibody* | *Company* | *1° or 2°* | *Anti-* | *Concentration* | *Dilution* |
| --- | --- | --- | --- | --- | --- |
| Cytokeratin 8/18 | Novus Biologicals (Centennial, CO, USA) | 1° | Mouse | 200 µg/ml | 1:100 |
| Vimentin | Invitrogen (Thermo Fisher Scientific, Carlsbad, CA, USA) | 1° | Rabbit | 1.04 mg/ml | 1:100 |
| Glycodelin A | Bio-Techne (Minneapolis, MN, USA) | 1° | Rabbit | 1.5 mg/ml | 1:100 |
| Goat Anti-Mouse IgG (H+L)*, Alexa Fluor Cf®568 | Invitrogen (Thermo Fisher Scientific, Carlsbad, CA, USA) | 2° | Mouse | 2 mg/ml | 1:350 |
| Goat Anti-Mouse IgG (H+L)*, Alexa Fluor Cf®488 | Invitrogen (Thermo Fisher Scientific, Carlsbad, CA, USA) | 2° | Rabbit | 2 mg/ml | 1:200 |
| Goat Anti-Rabbit IgG (H+L)* Alexa Fluor Cf®568 | Invitrogen (Thermo Fisher Scientific, Carlsbad, CA, USA) | 2° | Rabbit | 2 mg/ml | 1:200 |
| Goat Anti-Rabbit IgG (H+L)*, Alexa Fluor Cf®488 | Invitrogen (Thermo Fisher Scientific, Carlsbad, CA, USA) | 2° | Rabbit | 2 mg/ml | 1:200 |

* **heavy (H)** and **light (L)** chains of IgG molecules
